# Measuring and classifying IP usage scenarios: a continuous neural trees approach

**DOI:** 10.1038/s41598-024-55750-x

**Published:** 2024-03-01

**Authors:** Zhenhui Li, Fan Zhou, Zhiyuan Wang, Xovee Xu, Leyuan Liu, Guangqiang Yin

**Affiliations:** 1 University of Electronic Science and Technology of China, Chengdu, 610054 China; 2 Kash Institute of Electronics and Information Industry, Kashi, 84400 China

**Keywords:** Computer science, Software

## Abstract

Understanding user behavior via IP addresses is a crucial measure towards numerous pragmatic IP-based applications, including online content delivery, fraud prevention, marketing intelligence, and others. While profiling IP addresses through methods like IP geolocation and anomaly detection has been thoroughly studied, the function of an IP address—e.g., whether it pertains to a private enterprise network or a home broadband—remains underexplored. In this work, we initiate the first attempt to address the IP usage scenario classification problem. We collect data consisting of IP addresses from four large-scale regions. A novel continuous neural tree-based ensemble model is proposed to learn IP assignment rules and complex feature interactions. We conduct extensive experiments to evaluate our model in terms of classification accuracy and generalizability. Our results demonstrate that the proposed model is capable of efficiently uncovering significant higher-order feature interactions that enhance IP usage scenario classification, while also possessing the ability to generalize from the source region to the target one.

## Introduction

An Internet Protocol (IP) address is a unique identifier assigned to devices interfacing with the Internet, functioning as a means of personal identifiable information and location-based addressing. The detailed examination and analysis of IP addresses, which includes the investigation of risk behaviors associated with these addresses across various dimensions, is collectively referred to as IP Address Profiling (IAP)^1,2^, the practice of mapping IP addresses to their respective geographical locations. This pivotal step serves as the foundations for a multitude of downstream applications, which range from targeted marketing and fraud prevention to restricted content delivery and network attack detection^3–7^.

In the present study, we investigate a new research problem in IAP – IP usage scenario classification (IPUSC)—aiming at predicting the roles of IP address owners, such as data centers and home broadband, by scrutinizing the network attributes and behaviors correlated with IPs. This problem is important in various network-based applications and online services. Accurately measuring usage scenarios can enhance system legality and authenticity, assist companies in mitigating fraudulent risks, improve service management, and bolster defenses against online attacks. By probing into the application scenario of IP addresses, advertising companies and demand-side platforms can sift out bot-generated IPs, optimizing resource allocation strategies. This optimization minimizes online advertising costs directed at non-human traffic, enhances the effectiveness of advertisement delivery, and maximizes return on investment. Such an analysis can also aid in identification of so-called “wool parties”—these are farms that manipulate application rankings and search outcomes via advertisement fraud, used in tandem with other verification technologies during login, transaction, and payment processes. Furthermore, predicting IP usage scenario is beneficial in managing financial credit risk, as it enables the identification of fraudulent activities and high-risk users, monitoring business credit risks, and verifying whether transactions are conducted by bots controlled by malicious entities^8,9^.

Specifically, we delve into a new research question: *Can IP usage scenarios be effectively classified?* To answer this question, we extensively extract IP-related features through active Internet measurements (e.g., traceroute, GPS, and Wi-Fi) and several open databases (e.g., WHOIS, DNS, and IP geolocations). We design a novel deep continuous neural tree-based ensemble model, which takes the advantages of both deep learning and ensemble models for classifying IP addresses into one of the four usage scenarios: home broadband, private enterprise, cellular network, and data center. The exploration of this classification method adds a new layer of protection and understanding to the ongoing dialogue about the dynamics and nuances of IP address utilization.

To the best of our knowledge, this is among the first work towards proposing an effective and efficient method to address the IPUSC problem. Our approach leverages rich network measurements and deep continuous neural trees to capture various explicit and implicit IP features and model their complex interactions. We employ differentiable boosted decision trees^10,11^ to learn interpretable feature transformations and facilitate model differentiability into the feature splitting and decision tree routing. Moreover, our method stacks multiple layers of ensemble trees through deep continuous neural networks for learning decision rules. Rather than directly using neural networks for stacking discrete layers^12^ that may undermine the continuous feature learning, we introduce neural ordinary differentiable equations^13^ to consider the complex dependencies between consecutive layers.

To sum up, this study makes the following contributions:

- We formally formulate a novel and useful perspective on IP address profiling—exploring the usage scenario of an IP address – which can benefit many online services such as risk management and precise advertising.
- We collect and present large-scale benchmark datasets for IP usage scenario classification, which consists of a large volume of IP addresses as well as a wide range of corresponding categorical and numerical features organized in a format of tabular.
- We propose a novel deep continuous neural trees approach to explore the IP scenario tabular data. Our model takes both the advantages of differentiable decision trees and deep neural networks, bridging the gap between continuous feature learning and discrete neural ensembles.
- Extensive experiments conducted on four large-scale benchmark datasets demonstrate the effectiveness of our model on classifying IP usage scenarios in comparison to strong baselines. Our model can precisely fit the IP assignment rules crossing ISPs, showing superior transferring capability without significant performance degradation. This is especially useful for regions with data limitations or restrictions. Our method may benefit various online services such as risk management and fraud prevention.

## Related work

### IP profiling

Understanding user behaviors behind IP addresses is important for many real-world applications, e.g., fraud detection, malicious behavior detection, and precise positioning. IP address profiling (IAP) aims to represent IP hosts from the measured network traffic data and summarize communication behaviors and usage patterns. Many IAP tasks, including IP geolocation^2,14,15^, network traffic classification^16^, and host behavior profiling^17^, have been studied in literature. For example, IP geolocation maps an IP address to a physical location such as a country, a city, or even a street, which has been extensively studied in the community due to its importance on online fraud prevention and personalized content delivery^18,19^. However, to our knowledge, little effort has been done towards identifying the IP usage scenarios, which try to figure out the types of IP addresses. IP scenario prediction can be used as a preliminary for IAP tasks. For example, it can help risk control for credit card business by analyzing users’ logins and transaction IP addresses. Zhou et al.^20^ formulated the IP usage scenario classification problem and introduced a benchmarking dataset. Our work builds upon this groundwork and proposes a novel deep continuous neural trees approach that outperforms strong baselines. We also conducted model generalization experiments that showcase the proposed model has better transferring capability across different regions.

### Tabular data learning

Tabular data consist of rows for instances (e.g., IP blocks) and columns for features (e.g., the port and domain name). Tree-based ensemble models are widely used for learning informative signals and complex feature interactions from tabular data. They are both efficient and effective, and their predictions are highly interpretable. Nevertheless, tree-based models require heavy feature engineering and do not support representation learning for end-to-end training.

Recently, there is a growing interest in combining the advantages of deep neural networks and ensemble decision trees for learning feature interactions in tabular data^12,21–26^. For example, Autocross^22^ is an automatic feature crossing method designed for tabular data mining and classification which is especially suitable for capturing considerable categorical feature interactions. NON^26^ is a deep tabular network model by adding an auxiliary classifier to each layer of networks. It leverages three different neural networks to exploit the intra-field information and explore the non-linear feature interactions for tabular data classification.

## Data and problem

**Figure 1:**
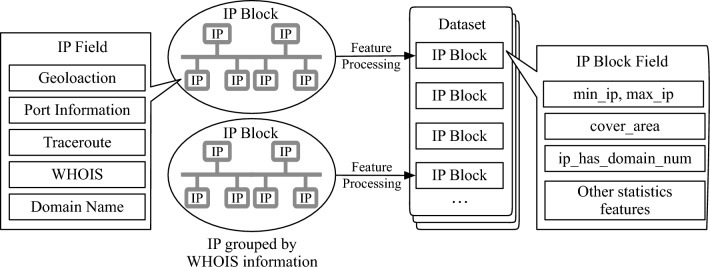
Overview of data acquisition.

Now we describe the details of data and features, and then formally define the IP usage scenario classification problem. An illustration of data acquisition is depicted in Fig. 1.

### IP block construction

During data inspection, we find that continuous IP addresses are usually used in the same scenario. Therefore, we consider IP blocks rather than individual IP addresses when predicting usage scenarios. Besides, features of an IP block are more stable and can be easily understood than individual IP addresses.

An IP block is composed of a sequence of continuous IPs. In our data, segmenting IPs into blocks is based on the smallest IP subnetwork division in the WHOIS database. If the number of IPs in a subnetwork is more than 256, this IP block continues with another 256 IPs.

### Feature extraction

Extracting informative IP block features is the key step for IP usage scenario classification problem. Specifically, we focus on the following groups of features. The list of features is shown in Table 1.

1. **Geographic location information**: We collect IP geolocation information from devices GPS signals. In an IP block, IPs with geolocation records are denoted as landmarks. Other types of geolocation features extracted from landmarks are also considered, such as landmark ratios and administration divisions. Meanwhile, since terrains and social factors may affect IP distributions, we include the following new features: area, area GDP, population, and population density.
2. **Routing information**: We use the remote traceroute method^27^ to obtain IP routing messages in a block and record *intermediate routing IPs*, *round-trip time*, and *reachable results*. Based on the recorded data, we select two most indicative features: the proportion of reachable IPs and the proportion of IPs appearing in a routing path. The rationale behind this two features is that IPs along traceroute paths usually belong to the data centers or private enterprises. In contrast, if a majority of IPs in a block cannot be accessed, they are more likely to be assigned to home broadband or cellular networks.
3. **Port information**: We deploy the network scanning tool ZMap^28^ to scan and record the opening status of reserving ports, e.g., 80/443 used for HTTP/HTTPS, 21 used for FTP, and 22 used for SSH. For example, many 80/443 ports used by conventional web services are opened in private enterprises and data centers, while their usages are very limited for cellular networks and home broadband. Otherwise, port 53 used by DNS is more likely to appear in data centers.
4. **Domain and registration information**: We take domain names and registration information into account by accumulating abundant IP-domain data in DNS. For example, the number of domain ownership in data centers and private enterprises is higher than that in home broadband and cellular networks.

**Table 1:** List of features used in this work.

Group	# Features	Features
Geolocation	20	Number of IPs, number of landmarks, ratio of landmarks, average number of landmark history locations, landmark covered area radius, number of landmark covered districts, number of landmark covered cities, number of landmark covered provinces/states, average of the ratios of every landmark history covered area to the block covered area, length of IP block prefix (e.g., the 24 in 192.168.0.1/24), block province/state, block province/state area, block province/state GDP, block province/state population, block province/state population density, block city, block city area, block city GDP, block city population, block city population density.
Traceroute	4	Number and ratio of reachable IPs, number and ratio of IPs appearing in a routing path.
Port	16	Numbers and ratios of alive port for port 80, port 443, port 21, port 22, port 23, port 53, and ports for email services (including port 25, port 465, port 143, port 993, port 110, and port 995), number of ICMP alive IPs, ratio of ICMP alive IPs.
Domain	3	Average number of block IP main domain names, number of IP domain names, ratio of IP domain names.
WHOIS	3	Number of WHOIS IPs (e.g., 65536 and 32768), WHOIS registration netname, WHOIS registration organization name.

### IP usage scenario classification problem definition

Now we define the problem studied in this paper. Given a set of IP block features, which is consisted of 46 independent variables. We aim to build a data-driven model that classifies an IP block into one of the four typical IP usage scenarios: *Home Broadband*, *Private Enterprise*, *Cellular Network*, or *Data Centers*.

## Methods

This section presents the overall framework for addressing the IPUSC problem.

### Tree-based classification model

Since real-world IP scenario assignments are usually assigned by flexible rules, we choose tree-based models that follow consistent divide-and-conquer rules and can provide interpretable predictions. The tabular data we studied contain a large number of numerical and categorical features. Tree-based methods learn tabular data via a series of boosting models such as XGBoost^29^, LightGBM^30^, or CatBoost^31^. However, most of them are limited to decision trees whose constructions have unconstrained rules. Once the training process finishes, the decision rules will not change. They can fit data efficiently but may end up with overfitting issues and suboptimal classification performance.

To overcome these issues, we propose a novel tree-based neural network named *ODTSR* that can interactively handle tabular data with greater flexibility. Specifically, we use Oblivious Decision Tree (ODT)^10,11^ as the basic learning architecture. It is similar to a regular decision tree but is constrained by the same feature and splitting function in all decision nodes at the same depth. These constraints not only enhance our model’s generalization capability but also improve model’s efficiency as it allows parallel computing with independent splits—regular decision trees, in contrast, requiring sequentially splits.

**Figure 2:**
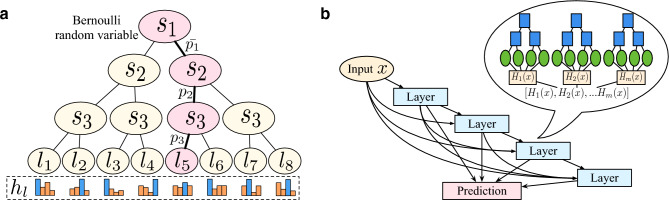
Method overview. (**a**) Illustration of an ODTSR Tree. (**b**) The architecture of multiple-layer ensembles.

The main drawback of tree-based approaches is that they are based on a divide-and-conquer strategy that does not allow end-to-end optimization and local optimization. To make the ODT differential, we introduce a stochastic routing^32^ into ODT and propose a novel model called ODTSR–ODT with Stochastic Routing. As illustrated in Fig. 2 (left panel), there are a set of intermediate nodes and leaf nodes. Different from the decision nodes of traditional decision trees that simply conduct routing by a binary number, the node routing directions in our proposed ODTSR are the output of a random variable, which provides feasibility for global optimization. Moreover, the split in traditional decision trees is determined by the Heaviside function. To make the tree output differentiable, we replace the split Heaviside function by a Bernoulli random variable with mean $s_d(z;\Theta )$, where *z* is a specific feature of an IP block, $\Theta$ is a learnable parameter, and function $s_d (\cdot )$ is defined as:

1 $$\begin{aligned} s_d(z;\Theta )=\sigma (\Theta ^\intercal z), \end{aligned}$$

where $\sigma$ is a nonlinear activation function (e.g., sigmoid here). Each leaf node maintains a class-label distribution, and each $h_l\in \mathbb {R}^4$ is the probability of each IP block belongs to a specific scenario. At last, the prediction *H* of the ODTSR is the averaged probabilities of all leaves:

2 $$\begin{aligned} H [x,\Theta ,p]=\sum _{l \in L} h_l p_l(x|\Theta ), \end{aligned}$$

where $p_l(x|\Theta )$ is the probability that sample *x* reaches leaf *l*:

3 $$\begin{aligned} p_l(x|\Theta ) = \prod _{d\in D} s_d(x;\Theta )^{\mathbbm {1} (l\in L_{\textrm{left}})}(1-s_d(x;\Theta ))^{\mathbbm {1} (l\in L_{\textrm{right}})}. \end{aligned}$$

Here $\mathbbm {1}(\cdot )$ is an indicator function, *D* denotes all decision nodes, $L_{\textrm{left}}$ and $L_{\textrm{right}}$ are the sets of decision nodes that go to left or right in the routing, respectively. Please refer to Fig. 2 (left panel) for an intuitive illustration for the path $(S_1, S_2, S_3, l_5)$.

### Deep tree ensembles

Now we have defined ODTSR to make the decision trees differentiable and follows an end-to-end structure, which enables parameter updating via backpropagation. However, since IP scenario data contains a large number of complex features, a single-layer of ODTSR may not be able to accurately explore and capture the intricate correlations and interplays among IP scenario features.

To overcome this hurdle, we introduce a deep tree ensemble technique inspired by the recent advances in bridging deep learning and gradient-based decision trees^12^, which have shown promising performance on learning tabular data. Specifically, there are *m* trees in each layer of the neural networks whose output is composed by the concatenation of all tree predictions $H^k = [H^k_1(x),H^k_2(x),...,H^k_m(x)]$, where $H^k$ denotes *k*-th layer output. In order to realize a deep network, the architecture is designed as a sequence of *K* layers, as shown in Fig. 2 (right panel), each layer takes sample *x* and the concatenation of all previous layers as its input. The relationship between each layer can be described as:

4 $$\begin{aligned} H^k = x + g^k(H^{k-1};\Theta ), \end{aligned}$$

where $g^k(\cdot )$ is ODTSR function at the *k*-th layer. In this way, the deep neural ensemble model can learn both shallow and deep decision rules while also capturing the interactions among IP blocks.

This deep structure is straightforward to motivate representation transformation, however, its layers have many different parameters that are hard to be optimized. Fortunately, the neural ODE^33^ implies the existence of an optimal network, which allows us to build adaptive deep layers. Following this idea, we transform the deep structure into a continuous form and use single ODTSR function to describe the evolution:

5 $$\begin{aligned}&\frac{d({H}^k)}{d k} = \text {ODTSR}(k, {H}^k+x; \Theta ), \end{aligned}$$

6 $$\begin{aligned}&H^k = H^{k-1}+ \int _{k-1}^{k} \text {ODTSR}( k^{\prime } , H^{k^{\prime }}+x;\Theta )dk^{\prime }, \end{aligned}$$

where we treat the ODTSR as an ODE block to model continuous layers and obtain representations of each layer with single-layer parameters. To solve the ODEs efficiently, we employ the fourth-order Runge–Kutta method^34^, which has higher precision than a simple Euler method:

7 $$\begin{aligned}&\textbf{R}_1 = \text {ODTSR}(k, H^{k^{\prime}}+x)\ , \end{aligned}$$

8 $$\begin{aligned}&\textbf{R}_2 = \text {ODTSR}(k+1/2, H^k+\textbf{R}_1/2+x)\ , \end{aligned}$$

9 $$\begin{aligned}&\textbf{R}_3 = \text {ODTSR}(k+1/2, H^k+\textbf{R}_2/2+x)\ , \end{aligned}$$

10 $$\begin{aligned}&\textbf{R}_4 = \text {ODTSR}(k+1, H^k+\textbf{R}_3+x)\ , \end{aligned}$$

11 $$\begin{aligned}&\int _{k}^{k+1}H(k^{\prime}, H^{k^{\prime}}+x;\Theta )\ dk^{\prime} =\frac{1}{6}(\textbf{R}_1 + 2\textbf{R}_2 + 2\textbf{R}_3 + \textbf{R}_4)\ , \end{aligned}$$

where $\textbf{R}_1, \textbf{R}_2$, $\textbf{R}_3$ and $\textbf{R}_4$ denote the derivative at the beginning, midpoint, and end of the interval. In this way, we approximate the integration with multi-step discrete processes.

The final prediction of the model is obtained by averaging outputs from all layers:

12 $$\begin{aligned} Q(x|\Theta )=\frac{1}{K}\sum _{k=1}^{K}H^k, \end{aligned}$$

where $H^k \in \mathbb {R}^{\left| c\right| }$ is the output of the *k*-th layer and $\left| c\right|$ is the number of classes.

### Training

We train our model via mini-batch SGD, which increases the convergence stability on the premise of reducing the computational cost. As for the optimizer, we use the method recommended by Ma et al.^35^ for efficiency. In terms of the optimization objective, considering that the output of each layer is a probability vector, we choose traditional cross-entropy loss that is usually employed in classification:

13 $$\begin{aligned} {\mathscr{L}} = -\frac{1}{|B|}\sum _{(x,y)\in B}\ln {\sum Q(x)\circ y}, \end{aligned}$$

where $\circ$ denotes the Hadamard product, *B* denotes the set of a mini-batch.

## Experiments

In this section, we first describe the experimental settings including datasets, baselines and metrics. Then we report experimental evaluation results on IP usage scenario classification.

### Experimental settings

#### Data

We evaluate our proposed method using the IP data collected from four regions: Shandong, Sichuan, and Chongqing City from China, and Illinois State from USA. We use 46 distinct IP-related features. The data statistics of four regions are shown in Table 2. For each region, we use 60% IP blocks for training, 20% for validation, and rest 20% for test.

**Table 2:** Descriptive statistics of datasets.

Region	IP Block	IP address	Area ($km^2$)	Population (M)
Sichuan	30,029	6,999,780	481,400	83.41
Shandong	67,443	12,731,730	153,800	100.47
Chongqing	18,719	3,304,308	82,300	31.02
Illinois	86,187	2,549,476	149,997	12.67

**Table 3:** Performance comparison on the IP scenario prediction.

Region	Sichuan			Shandong			Chongqing			Illinois		
Metric	Precision	Recall	Auc	Precision	Recall	Auc	Precision	Recall	Auc	Precision	Recall	Auc
SVM	0.8315	0.8735	0.9705	0.9560	0.9289	0.9916	0.9132	0.8956	0.9819	0.8977	0.8183	0.8902
BN	0.6112	0.6818	0.8944	0.8071	0.8074	0.9765	0.7492	0.8039	0.9524	0.4413	0.5613	0.8980
LDA	0.7719	0.7872	0.9553	0.7927	0.8768	0.9810	0.8186	0.8646	0.9717	0.5902	0.8816	0.9554
RF	0.8646	0.8159	0.9771	0.9614	0.9188	0.9936	0.9541	0.8602	0.9904	0.9852	0.5734	0.9250
XgBoost	0.8767	0.8683	0.9732	0.9548	0.9375	0.9947	0.9470	0.9273	0.9913	0.9851	0.8922	0.9708
CatBoost	0.8746	0.7616	0.9226	0.8710	0.9412	0.9805	0.7630	0.7711	0.9464	0.9216	0.7234	0.9003
TabNet	0.8425	0.8143	0.9623	0.9489	0.9275	0.9878	0.9423	0.8952	0.9757	0.8834	0.6128	0.9109
NON	0.7958	0.8483	0.9664	0.9274	0.9172	0.9917	0.9246	0.9152	0.9790	0.9346	0.7415	0.9303
AutoInt	0.8210	0.7704	0.9591	0.9535	0.9358	0.9926	0.9513	0.9006	0.9820	0.9619	0.8896	0.9661
NODE	0.8443	0.8147	0.9762	0.9601	0.9165	0.9904	0.9525	0.8591	0.9901	0.9843	0.5721	0.9239
ODTSR	0.8997	0.8759	0.9822	0.9629	0.9458	0.9954	0.9558	0.9368	0.9922	0.9861	0.9012	0.9876

#### Baselines

We evaluate our model against the following baseline methods that can be grouped into three categories: general machine learning-based, ensemble learning-based, and deep neural networks-based models.

- **Machine learning approaches**: Support Vector Machine (SVM)^36^, Bayesian Networks (BN)^37^, and Linear Discriminant Analysis (LDA).
- **Ensemble learning approaches** that combine several weak supervision models: Random Forest (RF), XGBoost^29^, and CataBoost^31^.
- **Deep learning based approaches**: 1) TabNet^21^: an efficient and interpretable deep tabular data learning model, which takes the raw tabular data as input without any feature pre-processing; 2) Network on Network (NON)^26^: a deep tabular data classifier for intra-field and non-linear feature interaction learning; 3) AutoInt^21^: an automatic feature interaction learning model using self-attentive neural networks^38^. and 4) NODE^12^: an ensemble tabular learning model that combines oblivious decision forests with dense residual networks^39^.

For all methods including ours, we tune model parameters using the validation data and report the best results on test set. We use the following three metrics: precision, recall, and area under the ROC curve (AUC). Precision is the fraction of relevant IP blocks among the retrieved samples, while recall is the fraction of the total amount of pertinent IP blocks that were actually retrieved. AUC is computed based on the relative ranking of all IP blocks’ prediction probabilities, which is not impacted by any simple scaling of predictions. As a multi-class classification problem, we average all confusion matrices to obtain the final results.

**Figure 3:**
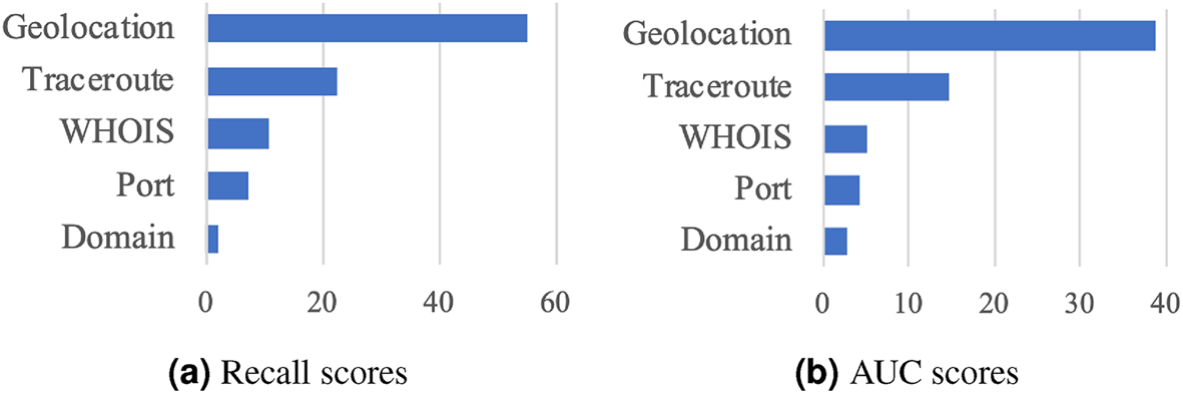
Illustration of feature importance.

**Table 4:** Model’s generalization capability between different regions.

Region	Sichuan $\rightarrow$ Chongqing			Chongqing $\rightarrow$ Sichuan			Shandong $\rightarrow$ Sichuan			Shandong $\rightarrow$ Chongqing		
Metric	Precision	Recall	Auc	Precision	Recall	Auc	Precision	Recall	Auc	Precision	Recall	Auc
SVM	0.6714	0.6725	0.8885	0.7993	0.7457	0.9382	0.8671	0.7530	0.9390	0.9114	0.7777	0.9645
NB	0.5641	0.5655	0.8650	0.6524	0.6590	0.8861	0.6726	0.6167	0.8928	0.7575	0.6547	0.9270
LDA	0.6809	0.6412	0.8208	0.6621	0.6417	0.8701	0.6606	0.5824	0.8878	0.8036	0.6134	0.9258
RF	0.6246	0.7799	0.9377	0.7709	0.7059	0.9106	0.8777	0.7098	0.8928	0.8930	0.7984	0.9632
XgBoost	0.6051	0.7543	0.9376	0.7360	0.6628	0.9118	0.8770	0.7065	0.9431	0.9001	0.8125	0.9468
CatBoost	0.6137	0.7697	0.9038	0.6136	0.4891	0.8345	0.7400	0.6616	0.8387	0.7589	0.7403	0.8876
TabNet	0.6336	0.7797	0.9158	0.7326	0.6936	0.9054	0.8063	0.7327	0.9097	0.8261	0.7920	0.9349
NON	0.6838	0.7384	0.8825	0.7097	0.7264	0.9041	0.8096	0.7639	0.9191	0.8013	0.8164	0.9267
AutoInt	0.5887	0.7402	0.8250	0.6802	0.6232	0.7333	0.8618	0.6741	0.7856	0.8384	0.7623	0.8212
NODE	0.6315	0.7778	0.9145	0.7321	0.6920	0.9034	0.8051	0.7319	0.9025	0.8245	0.7911	0.9335
ODTSR	0.7295	0.8042	0.9462	0.8409	0.7714	0.9444	0.8817	0.7679	0.9582	0.9322	0.8654	0.9763

**Figure 4:**
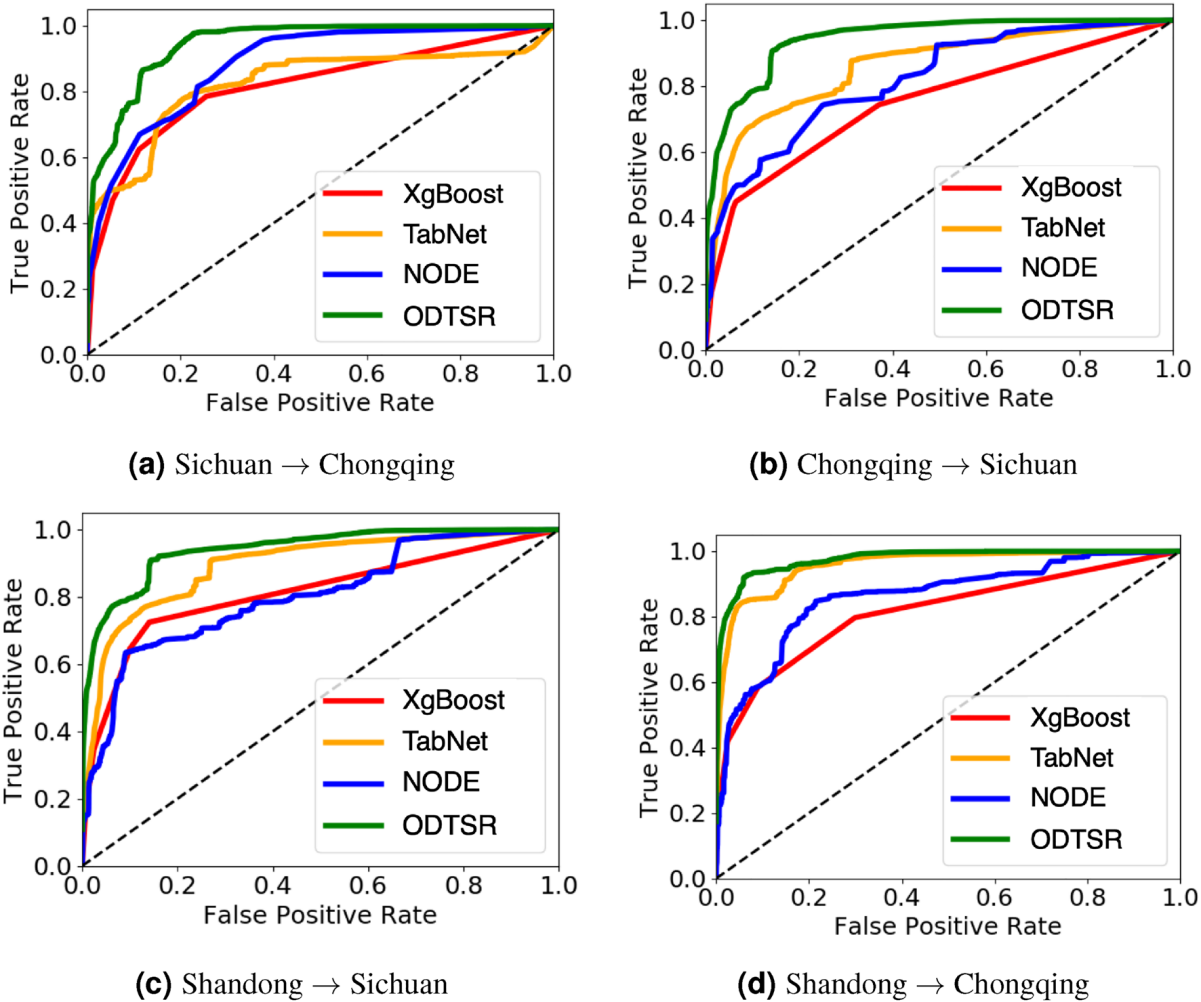
Performance evaluation on the models’ ability to fit the general IP assignment rules in different regions.

### Experimental results

We now report and discuss experimental results from four aspects: main comparison results, influence of features, model generalization, and parameter sensitivity.

#### Performance comparison

The overall performance evaluation of all methods are reported in Table 3, where paired *t*-test was performed for statistical significance ($p < 0.001$). We can see that our model achieves the best IP usage scenario performance across all metrics on four datasets. We have the following observations. *(a)* Traditional machine learning approaches (SVM, BN, and LDA) did not show comparable performance since they cannot capture complex dependencies among features. *(b)* Ensemble decision trees (RF, CatBoost, and XgBoost) offer non-trivial performance improvements due to their superior learning ability to fit the underlying decision manifolds and boost performance with approximate hyperplane boundaries, indicating that extracting complex decision rules from tabular data are important for IPUSC problem. *(c)* Three deep learning-based approaches have comparable performances compard to ensemble-based methods. They can efficiently encode multiple data types like numerical and categorical features along with the tabular data in an end-to-end manner, reducing the effort for hand-crafting features. *(d)* NODE did not bring additional improvements, Although NODE takes the advantages of both decision trees and neural networks, it does not bring additional improvements. This may be explained by the differentiable splitting functions are not well fitted with the discrete residual networks. In contrast, our method considers the continuous neural networks and learns continuously differentiable information flow in the consecutive neural layers and, as a result, smoothing the feature interactions for IP classification.

Interestingly, all models perform relatively well on Shandong, Chongqing, and Illinois regions but worse on Sichuan region. We speculate this is due to the topography differences among these regions. For example, Shandong’s population density is much higher than Sichuan, which implies that IP geographical distributions are much closer in Shandong than Sichuan, and the IP block discrimination task is easier for the Shandong region.

#### Influence of features

Recall that there are five groups of 46 features. To better understand their influence on IPUSC task, we conduct an ablation study to examine each group’s contribution to overall classification performance. Specifically, we shuffle the attributes of samples (IP blocks) in a group to observe the performance change, which could effectively reflect the relative importance of a specific group, e.g., a group’s influence is trivial if the result does not significantly changed. Figure 3 depicts the importance of each group—averaged by the performance changes in four regions. We can see that geographical information, e.g., coverage, distributions, and the number of landmarks, play an essential role in IPUSC task. This result is intuitive since IP geolocation is a strong signal to distinguish different scenarios. We also note that the landmark data is very sparse for certain scenarios. For example, the ratios of landmarks in home broadband and cellular networks are around 70% and 34%, respectively, due to the widely used GPS-required apps in these two scenarios. In contrast, only 0.2% of data center scenario IP blocks have landmarks. Routing information and domain names are also useful for identifying IP usage scenarios, while registration (WHOIS) and available port information are relatively less important. This is because the registration information is too general to distinguish real IP usage scenarios. This finding indicates that IPUSC requires data-driven methods since openly available databases cannot provide accurate usage type information.

#### Model generalizability

We conduct transfer learning experiments to investigate models’ abilities to learn general IP assignment rules across different regions. Towards this goal, we train our model as well as baselines on a source region and test model’s performance on a target region. For example, *Sichuan* $\rightarrow$ *Chongqing* denotes that the model is trained on Sichuan and evaluated on Chongqing. Table 4 reports IPUSC transfer learning results. Besides, we show the ROC curves of two groups of separate transfer learning experiments in Fig. 4.

We can observe that all methods’ performances are degraded when transferring from the source region to the target region. Nevertheless, our model’s performance degradation is the least compared to baselines, which shows that our proposed model can better learn general IP usage rules across different regions. This trait of ODTSR is especially useful for regions with limited or restricted data. This result also suggests that the IP assignment of different IP management agencies or IP service providers may follow similar allocation rules that can be learned to enable in-depth analysis for many downstream tasks, e.g., targeted advertising, user behavior profiling, and “wool-party” detection.

**Figure 5:**
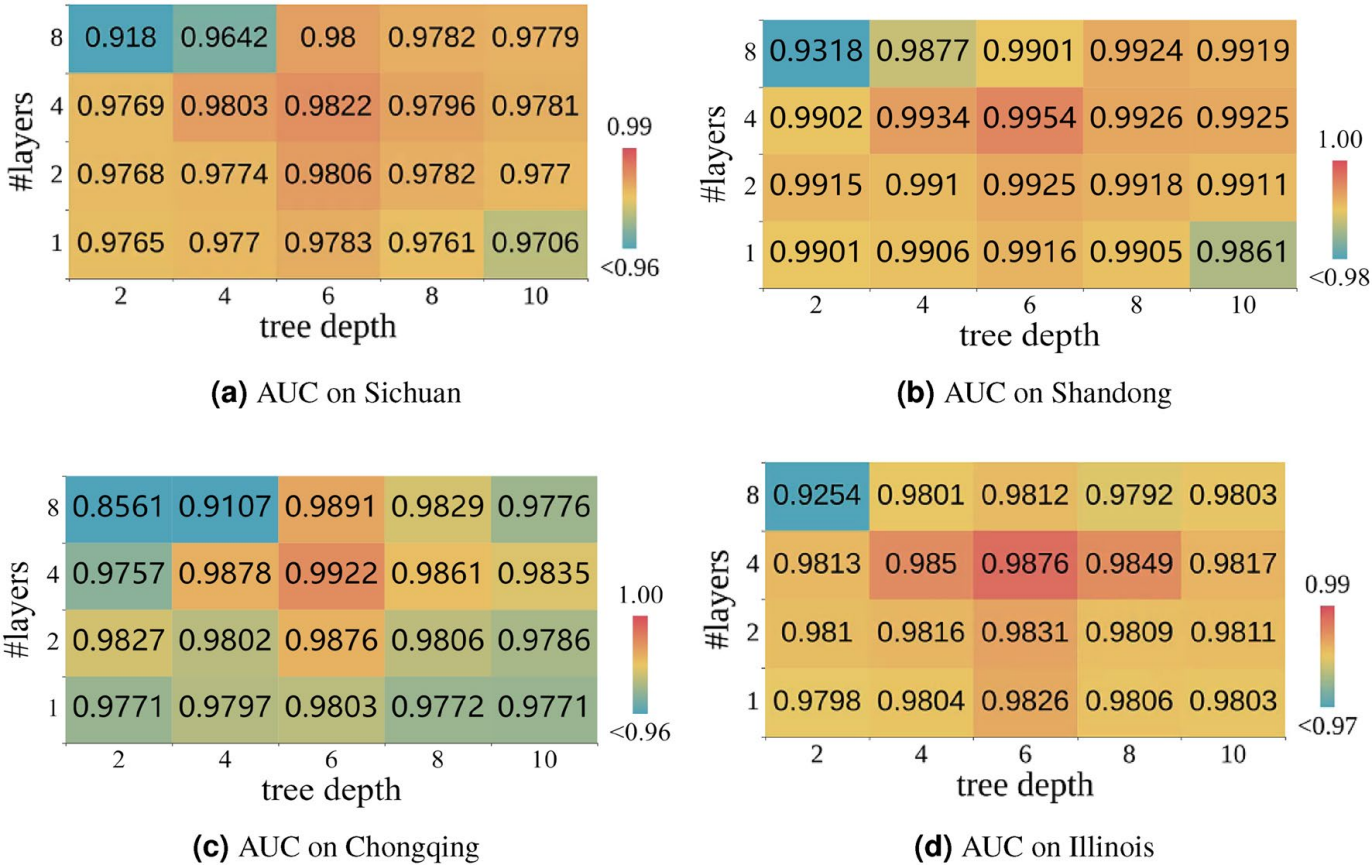
Influence of tree depth and network layers.

#### Parameter sensitivity

Our model has two critical parameters, i.e., the number of network layers and the tree depth. Figure 5 illustrates the influence of these two parameters, where we can see that a few network layers and moderate tree depth are enough for our model to achieve the best performance. Note that we did not observe significant overfitting problem if further increasing the network and tree depths, which is also the primary difficulty in combining deep learning and ensemble decision trees in the community^12,32^. The improvement attributes to our continuous deep ensemble learning method that models discrete ensembles with continuous layers, which bridges the gap between differentiable trees and discrete neural layers.

## Conclusion

In this work, we initiated the first attempt to study IP usage scenario classification, a new paradigm of IP address profiling that can benefit many downstream applications. We proposed a deep continuous ensemble learning approach based on differentiable decision trees and multi-layer neural networks. Our model stacks deep ensemble decision trees to capture both complex feature interactions and decision rules. Meanwhile, it incorporates numerical methods to solve the discrete stacking problem and provides continuous ensembles. Extensive experiments conducted on four regions demonstrate the effectiveness of our model on identifying IP usage scenarios by apprehending the IP address assignment rules. Moreover, the new designed model consistently outperforms both shallow ensemble learning methods and deep neural networks in IP-related tabular data learning. Empirical findings in this study may motivate future research on other IP-related network services such as traffic forecasting, IP geolocation, and network topology analysis.

## Data Availability

The datasets generated during and/or analysed during the current study are available from the corresponding author on reasonable request.
